# Positive syphilis serology contributes to intracranial stenosis in ischemic stroke patients

**DOI:** 10.1002/brb3.1906

**Published:** 2020-10-21

**Authors:** Lei Xiang, Tao Zhang, Biao Zhang, Chao Zhang, Wanzhen Cui, Wei Yue

**Affiliations:** ^1^ Department of Neurology Tianjin Key Laboratory of Cerebrovascular and Neurodegenerative Diseases Tianjin Huanhu Hospital Tianjin China; ^2^ Department of Intensive Care Unit Tianjin Huanhu Hospital Tianjin China; ^3^ Department of Clinical Laboratory Tianjin Huanhu Hospital Tianjin China

**Keywords:** intracranial, ischemic stroke, stenosis, syphilis

## Abstract

**Background and Purpose:**

The risk of ischemic stroke is increased among people living with syphilis infection; however, whether syphilis is an independently risk for stenosis is unclear. We investigated the clinical, laboratory, and vascular stenosis features of ischemic stroke patients living with positive syphilis serology to evaluate the role of syphilis in cerebral artery stenosis.

**Methods:**

The demographic, clinical characteristics, and the distribution of cerebral artery stenosis were compared between 668 syphilis‐positive and 785 syphilis‐negative ischemic stroke patients. Multivariate logistic regression analysis was performed to determine the degree and distribution of stenosis associated with positive syphilis serology and estimate the factors related to artery stenosis in the syphilis‐positive ischemic stroke patients.

**Results:**

Syphilis‐positive ischemic stroke patients were distinct from the nonsyphilis population, with a younger age, fewer women, and a different risk factor profile. Positive syphilis serology was independently associated with moderate stenosis (OR, 2.31; 95% CI 2.02–2.69; *p* = .003) and severe stenosis (OR, 6.15; 95% CI, 2.85–8.94; *p* < .001), mainly intracranial stenosis (OR, 1.49; 95% CI, 1.15–1.92; *p* = .002) rather than extracranial stenosis. Among stroke patients with positive syphilis serology, the higher RPR titer (OR, 1.18, 95% CI, 1.07–1.89 for RPR titer 1:16; OR, 5.16, 95% CI 2.99–8.89 for RPR titer > 1:32) and previous unknown or untreated syphilis (OR, 3.63; 95% CI, 2.72–4.03; *p* < .001) were the factors related to stenosis.

**Conclusions:**

Syphilis infection, especially when less well controlled, may play an important role in intracranial stenosis of ischemic stroke patients.

## Graphical abstract

Ischemic stroke patients living with positive syphilis serology show a tendency to be more prevalent in intracranial artery stenosis. Syphilis infection, especially when less well controlled, may play an important role in intracranial stenosis of ischemic stroke patients.

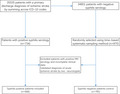

## 1 INTRODUCTION

As syphilis incidence continues to increase in many countries around the world, it remains a threat to public health and should not be overlooked (Peeling et al., 2017). Although the effectiveness of penicillin for the treatment of syphilis was well established, patients untreated during the earlier stages of syphilis are at risk of serious complications involving the central nervous system (CNS) (Chen et al., 2007; Tang et al., 2017). There have been many reported cases of syphilis patients suffering from a stroke, and syphilis is probably an important risk of stroke especially in young people (Cordato et al., 2013).

Recent work suggests that the risk of ischemic stroke is increased among people living with syphilis infection compared with uninfected individuals, and their clinical presentations of ischemic lesions in the same locations are generally similar (Liu et al., 2012). Therefore, misdiagnosis of syphilis‐related stroke is a common phenomenon, resulting in a lack of appropriate treatment for syphilis and further leading to more severe neurologic damage. Serologic screening for syphilis should be a routine part of the evaluation of stroke patients.

Syphilis could have a causal role in ischemic stroke, but there are relatively few epidemiologic data quantifying or qualifying associations of syphilis infection with stroke risk. The mechanisms by which syphilis infection has been linked to increased risk of stroke are still open to debate, and the most convincing one is vasculitis. Its main pathologic changes are extensive meningeal and perivascular inflammatory cell infiltration, intimal fibrosis, and hyperplasia, which further result in vascular stenosis or occlusion and secondary ischemic stroke (Weyand & Goronzy, 2013). Although here is growing evidence that duration of syphilis infection and its associated chronic inflammatory state may linked to subclinical vasculopathy (Flint et al., 2005), little is known about the distribution and related factors of cerebral artery stenosis in stroke patients with syphilis infection.

Since the routine screening of syphilis serology in stroke patients in our stroke center, the diagnostic rate of syphilis‐related stroke has been increasing. Thus, we performed a retrospective study to analyze the clinical, laboratory, and vascular stenosis features of ischemic stroke patients living with positive syphilis serology, estimate how they differ from the nonsyphilis ischemic stroke population. We also sought to determine the distribution of cerebral artery stenosis in syphilis‐positive patients and further explore the role of syphilis in cerebral artery stenosis.

## 2 METHODS

### 2.1 Study design and patients

**Figure 1:**
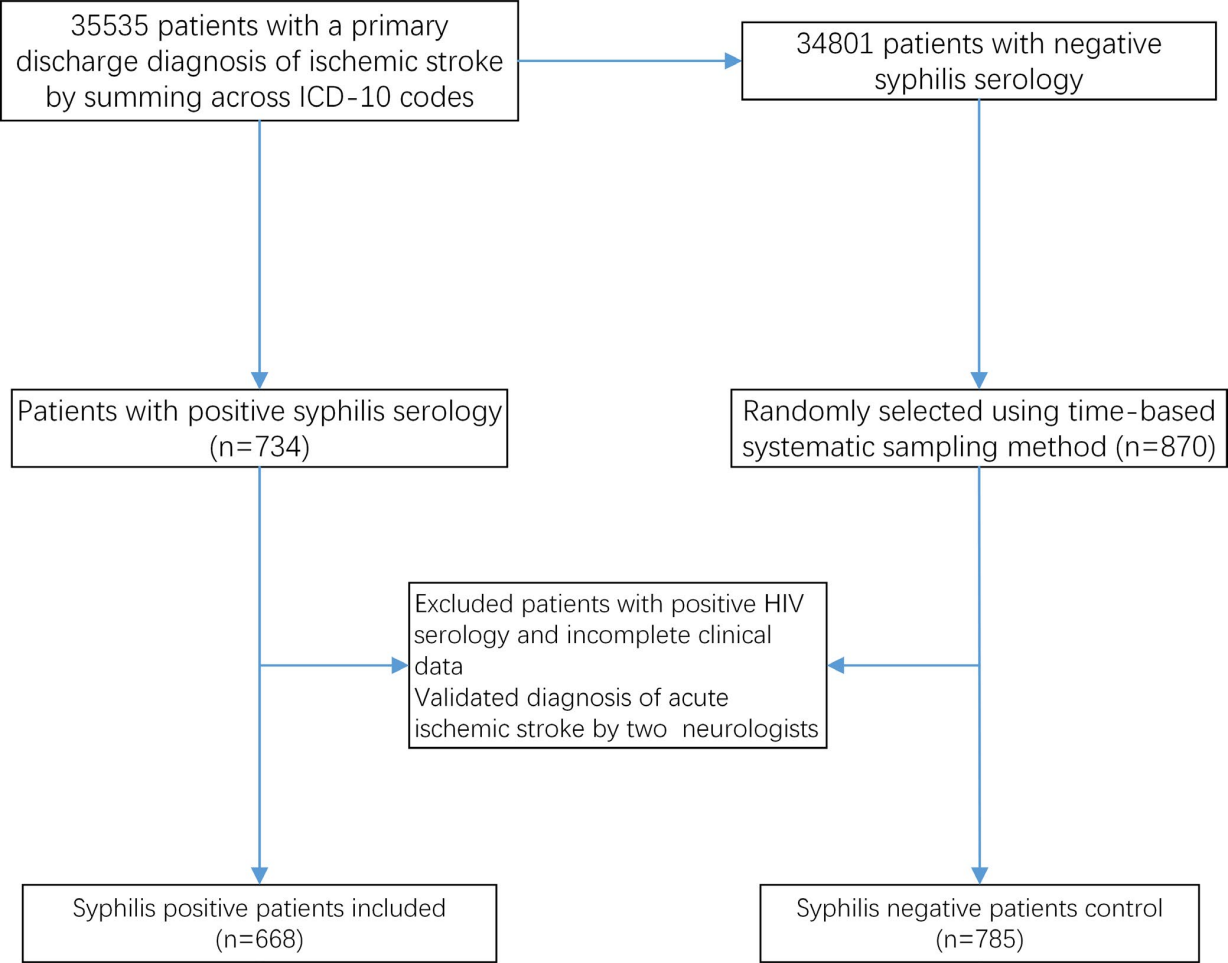
The flow chart of recruitment

We retrospectively viewed records of all hospitalized patients in our stroke registry from March 2016 to September 2018. Total number of ischemic stroke hospitalizations was 35,535 obtained by summing across ICD‐10 codes with a primary discharge diagnosis of ischemic stroke. Serological antibody tests for syphilis and HIV are routine at admission in our stroke center. The recruitment flow chart is shown in Figure 1. Patients with positive HIV serology and incomplete data were excluded. The negative syphilis serology samples were randomly selected from the 34,801 ischemic stroke patients with negative syphilis serology at a rate of 1:40 by time‐based systematic sampling method. The diagnosis of ischemic stroke was then validated by two board‐certified neurologists through medical record review, meeting the definition of acute ischemic stroke according to American Heart Association/American Stroke Association World Health Organization (Sacco et al., 2013). Finally, 668 patients with positive syphilis serology and 785 patients with negative syphilis serology were enrolled in this study. This study was approved by the Clinical Research Ethic Committee of Tianjin Huanhu hospital and performed in accordance with the ethical standards in the Declaration of Helsinki.

### 2.2 Clinical variables

Demographics, history of stroke risk factors, health‐related behaviors, and laboratory test results were abstracted from medical records. Individuals with positive syphilis serology were established through the documented detection of rapid plasma reagin (RPR) and T. pallidum particle agglutination (TPPA). Patients with negative TPPA serology were excluded. Past treatment for syphilis was determined from the medical record. Routine blood and biochemical tests, brain computed tomography, and/or magnetic resonance scan were performed in all ischemic stroke patients at admission. Stroke subtype was defined according to the TOAST classification (Goldstein et al., 2001), which includes 5 subtypes: large artery atherosclerosis (LAA), cardioembolism (CE), small‐vessel occlusion (SVO), stroke of other determined etiologies (SOE), and stroke of undetermined etiologies (SUE).

### 2.3 Carotid atherosclerosis

In all stroke subjects, high‐resolution ultrasound of the right and left carotid arteries was performed. The carotid artery intima–media thickness (CIMT) was measured off‐line from the media–adventitia interface to the intima–lumen interface in the near and far walls of the common carotid artery, 1–2 cm proximal to carotid bifurcation, and internal carotid artery at 12 carotid sites for both sides. The presence of plaque, defined as a focal structure that encroaches into the arterial lumen of at least 0.5 mm or 50% of the surrounding CIMT value, was also noted. The mean of CIMT was documented in each of these carotid segments and measured outside the portion of plaque if plaques were present in a given segment. Carotid atherosclerosis was defined as the presence of atherosclerotic plaques in any of the aforementioned arterial segments.

### 2.4 Cerebral arterial stenosis assessment

Extracranial arteries, including the common carotid artery (CCA), internal carotid artery (ICA), vertebral arteries (VA), and subclavian arteries (SA), were examined by carotid ultrasound. Intracranial arteries, including the middle cerebral artery (MCA), anterior cerebral arteries (ACA), posterior cerebral arteries (PCA), the terminal of the ICA (TICA), basilar artery (BA), and the intracranial segment of the VA, were examined by head MRA. Aortic arch to intracranial CTA was used to define stenosis of intracranial arteries in those patients where MRI was contraindicated. Different degrees of stenosis of these arteries were categorized as normal, mild (<50%), moderate (50%–69%), severe (70%–99%), and occlusion, respectively. All the patients with at least one of the above arteries having a degree of stenosis ≥ 50% were defined as stenosis. It should be noted that due to very low prevalence of ACA, PCA, and SA stenosis alone(Zhao et al., 2011), we did not analyze the stenosis of ACA, PCA, and SA into the final statistical analysis.

### 2.5 Statistical analyses

Continuous variables were presented as mean and standard deviation (mean ± *SD*), whereas categorical variables were presented as percentages. We compared demographic and clinical parameters between the two groups using Student's *t* test or Mann–Whitney test for continuous variables and chi‐squared or Fisher's exact test for categorical variables, as appropriate. Logistic regression analysis was first utilized to determine the association between stenosis and syphilis infection and analyze the factors associated with stenosis in syphilis‐infected stroke patients. We then used a multivariable model to estimate adjusted odds ratios (ORs) and 95% confidence intervals (CIs) after adjusting for demographics plus traditional vascular risk factors, including age, sex, smoking, alcohol consumption, low physical activity, obesity, hypertension, diabetes mellitus, coronary heart disease, and history of previous stroke. We used a *p*‐value < 0.05 considered statistically significant. All statistical analyses were performed with SPSS version 19.0.

## 3 RESULTS

The percentage of coexistent positive syphilis serology among ischemic stroke patients was about 1.88%. The baseline characteristics of the patients according to the syphilis serology are presented in Table 1. Compared with the syphilis‐negative patients, the syphilis‐positive patients were significantly younger (mean age, 60.1 ± 12.6 versus 63.0 ± 11.5; *p* < .001), more male (75.6% versus 67.5%; *p* = .001), and more likely to be alcohol drinker (32.9% versus 22.9%; *p* < .001). In contrast, other stroke risk factors, including diabetes, coronary heart disease, and previous stroke, were more common in patients who were syphilis‐negative (Table 1). There were no differences in smoking status, low physical activity, obesity, hypertension, or laboratory analysis including fasting glucose and cholesterol between two groups classified by syphilis serology. In terms of stroke subtypes, the incidence of large artery atherosclerosis was 31.1% in syphilis‐positive patients, which is significantly higher compared to 24.7% in syphilis‐negative group (*p* = .04). However, there were no significant differences in other etiological subtypes. In view of higher prevalence of large artery atherosclerosis, carotid atherosclerosis and vascular stenosis were further evaluated.

**Table 1:** The demographic and clinical characteristics of ischemic stroke patients according to syphilis serology

	Syphilis positive (*n* = 668)	Syphilis negative (*n* = 785)	*p*
Age (y)	60.1 ± 12.6	63.0 ± 11.5	<0.001
Male (*n*, %)	505(75.6)	530(67.5)	0.001
*Lifestyle*			
Alcohol drinker (*n*, %)	220 (32.9)	180 (22.9)	<0.001
Smoking (*n*, %)	254 (38.0)	285 (36.3)	0.514
Low physical activity (*n*, %)	115 (17.2)	141 (18.0)	0.832
Obesity (*n*, %)	93 (13.9)	125 (15.9)	0.083
*Medical history*			
Diabetes mellitus (*n*, %)	171 (25.6)	240 (30.6)	0.039
Coronary heart disease (*n*, %)	117 (17.5)	206 (26.2)	<0.001
Hypertension (*n*, %)	433 (64.8)	546 (69.6)	0.062
Previous stroke (*n*, %)	33 (4.9)	141 (18.0)	<0.001
*Laboratory analysis*			
Fasting glucose (mM)	6.1 ± 2.1	6.3 ± 2.4	0.132
LDL (mM)	2.9 ± 0.9	3.0 ± 0.8	0.067
HDL (mM)	1.0 ± 0.2	1.0 ± 0.3	0.127
hsCRP (mg/L)	1.95 ± 0.24	1.68 ± 0.37	<0.001
*Stroke subtype*			
LAA (*n*, %)	208 (31.1)	194 (24.7)	0.040
SVO (*n*, %)	217 (32.5)	268 (34.1)	0.637
CE (*n*, %)	71 (10.6)	97 (12.4)	0.360
SOE or SUE (*n*, %)	172 (25.7)	226 (28.8)	0.328
*Atherosclerosis characteristics*			
CIMT (mm)	0.75 ± 0.10	0.75 ± 0.11	0.768
Carotid artery plaque (*n*, %)	383 (57.3)	448 (57.1)	0.831
*Different degrees of stenosis*			
No (*n*, %)	360 (53.9)	460 (58.6)	0.080
Mild (*n*, %)	66 (9.9)	83 (10.6)	0.729
Moderate (*n*, %)	68 (10.2)	65 (8.3)	0.523
Severe or occlusion (*n*, %)	174 (26.0)	177 (22.5)	0.035
*Location of vascular stenosis* ^a^			
**Extracranial** (*n*, %)	70 (10.5)	74 (9.4)	0.538
CCA (*n*, %)	5 (0.7)	7 (0.9)	0.998
ICA (*n*, %)	43 (6.4)	52 (6.6)	0.916
Extracranial VA (*n*, %)	32 (4.8)	46 (5.9)	0.414
**Intracranial** (*n*, %)	189 (28.3)	180 (22.9)	0.022
ACA (*n*, %)	19 (2.8)	8 (1.0)	0.011
TICA (*n*, %)	10 (1.5)	15 (1.9)	0.687
MCA (*n*, %)	142 (21.3)	125 (15.9)	0.010
Intracranial VA (*n*, %)	40 (6.0)	57 (7.3)	0.345
BA (*n*, %))	23 (3.4)	35 (4.5)	0.349

Note

Values are expressed as the mean ± *SD* for a normal distribution and as the absolute number (percentage) for categorical variables.

Abbreviations: ACA, anterior cerebral artery; BA, basilar artery; CCA, common carotid artery; CE, cardioembolism; CIMT, carotid artery intima–media thickness; HDL, high density lipoprotein; hsCRP, hypersensitive C‐reactive protein; ICA, internal carotid artery; LAA, large artery atherosclerosis; LDL, low density lipoprotein; MCA, middle cerebral artery; SOE, stroke of other determined etiologies; SUE, stroke of undetermined etiologies; SVO, small‐vessel occlusion; TICA, terminal of internal carotid artery; VA, vertebral arteries.

^a^ Multiple occurrences possible.

The assessment of carotid atherosclerosis focused on CIMT and the presence of carotid atherosclerotic plaques in this study. There was no significant difference of carotid atherosclerosis between seropositive patients with seronegative ones (0.75 ± 0.10 versus 0.75 ± 0.11, *p* = .768 for CIMT; 57.3% versus 57.1%, *p* = .831 for plaque; Table 1). Atherosclerotic stenosis is the main cause of ischemic stroke. In total, the frequency and degree of cerebral artery stenosis were significantly increased in syphilis‐positive patients. Among the 668 seropositive ischemic stroke patients, 242 patients (36.2%) had at least one artery with a degree of stenosis ≥ 50%. The prevalence rate of severe stenosis or occlusion in syphilis‐positive patients was 26% which was notably higher than the rate in syphilis‐negative patients (22.5%, *p* = .035; Table 1). Results from univariate and multivariate logistic regression models for the association between stenosis and syphilis infection are shown in Table 2. After adjusting for demographics, lifestyle, and other ischemic stroke risk factors, the correlation between severe stenosis and positive syphilis serology was significantly enhanced (OR, 6.15; 95% CI, 2.85–8.94; *p* < .001; Table 2), and moderate stenosis was also independently associated with positive syphilis serology (OR, 2.31; 95% CI 2.02–2.69; *p* = .003; Table 2).

**Table 2:** Unadjusted and adjusted model for the association between stenosis and positive syphilis serology

Variables	Univariate Odds Ratio (95% CI)	*p*	Adjusted Model* Odds Ratio (95% CI)	*p*
**Degree of stenosis**				
No	1.00 (referent)		1.00 (referent)	
Mild	1.01 (0.71–1.44)	0.929	1.20 (0.82–1.73)	0.334
Moderate	1.22 (0.84–1.76)	0.285	2.31 (2.02–2.69)	0.003
Severe or occlusion	1.29 (1.06–1.66)	0.045	6.15 (2.85–8.94)	<0.001
**Location of vascular stenosis**				
**Extracranial**				
No	1.00 (referent)		1.00 (referent)	
Yes	1.12 (0.79–1.58)	0.499	1.09 (0.76–1.56)	0.616
CCA				
No	1.00 (referent)		1.00 (referent)	
Yes	0.83 (0.26–2.65)	0.766	1.03 (0.30–3.33)	0.997
ICA				
No	1.00 (referent)		1.00 (referent)	
Yes	0.97 (0.63–1.47)	0.891	0.86 (0.56–1.32)	0.504
Extracranial VA				
No	1.00 (referent)		1.00 (referent)	
Yes	0.80 (0.50–1.28)	0.371	0.79 (0.49–1.28)	0.348
**Intracranial**				
No	1.00 (referent)		1.00 (referent)	
Yes	1.32 (1.04–1.68)	0.019	1.49 (1.15–1.92)	0.002
ACA				
No	1.00 (referent)		1.00 (referent)	
Yes	2.84 (1.23–6.54)	0.014	2.83 (1.10–8.93)	0.016
TICA				
No	1.00 (referent)		1.00 (referent)	
Yes	0.78 (0.34–1.75)	0.549	1.50 (0.53–4.28)	0.440
MCA				
No	1.00 (referent)		1.00 (referent)	
Yes	1.42 (1.09–1.86)	0.009	5.38 (3.22–8.99)	<0.001
Intracranial VA				
No	1.00 (referent)		1.00 (referent)	
Yes	0.81 (0.53–1.23)	0.336	0.79 (0.41–1.51)	0.477
BA				
No	1.00 (referent)		1.00 (referent)	
Yes	0.76 (0.44–1.30)	0.328	1.18 (0.57–2.11)	0.648

Abbreviations: ACA, anterior cerebral artery; BA, basilar artery; CCA, common carotid artery; CI, confidence interval; ICA, internal carotid artery; MCA, middle cerebral artery; TICA, terminal of internal carotid artery; VA, vertebral arteries.

* Regression model adjusted for age, sex, smoking, alcohol consumption, low physical activity, obesity, hypertension, diabetes mellitus, coronary heart disease, and history of previous stroke.

The distribution of intracranial and extracranial artery stenosis in the ischemic stroke patients according to the syphilis serology is presented in Table 1. Intracranial carotid stenosis, but not extracranial stenosis, was more frequently observed in ischemic stroke patients with positive syphilis serology compared with syphilis‐negative group (28.3% versus 22.9%, *p* = .022; Table 1). We performed further multivariable binary logistic regression analysis and found that positive syphilis serology was independently associated with intracranial stenosis (OR, 1.49; 95% CI, 1.15–1.92; *p* = .002; Table 2), mainly MCA stenosis (OR, 5.38; 95% CI, 3.22–8.99; *p* < .001; Table 2) and ACA stenosis (OR, 2.83; 95% CI, 1.10–8.93; *p* = .016; Table 2) after adjusting demographics, lifestyle, and other risk factors for vascular stenosis.

**Figure 2:**
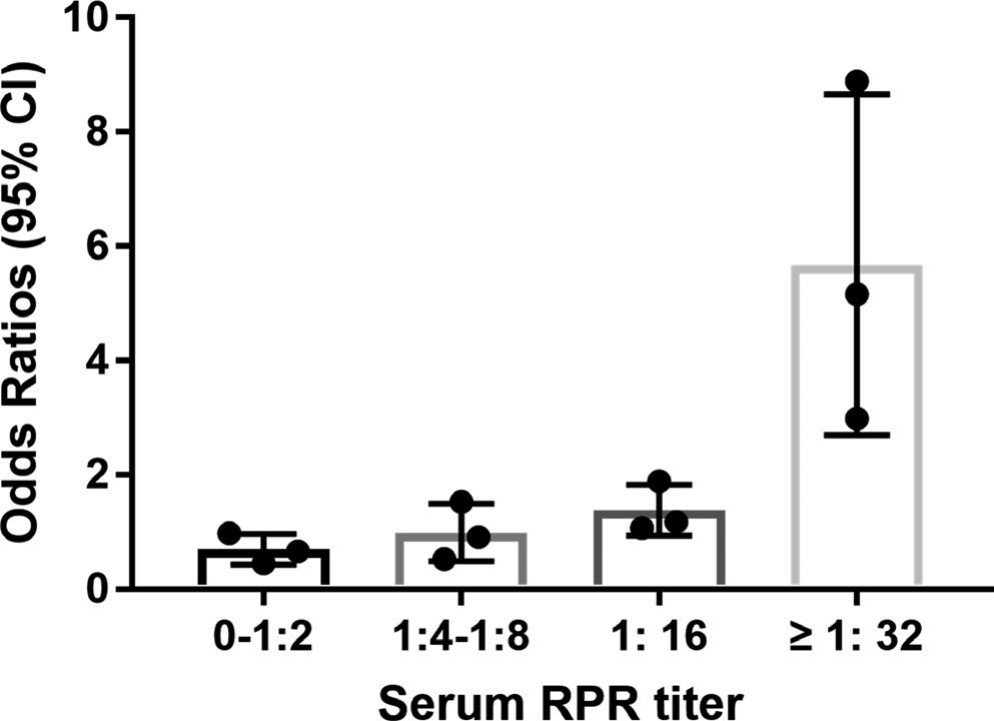
The trends of correlation between artery stenosis with serum RPR titer Odds ratios are shown according to the quartile of serum RPR titer. The odds ratios significantly increased with the increase of serum RPR titer (trend *p* < .001). Error bars indicate the 95% confidence interval of each value

In an analysis restricted to ischemic strokes with syphilis‐positive individuals, factors associated with artery stenosis are shown in Table 3. After adjusting for covariates, stenosis was independently associated with the higher RPR titer (OR, 1.18, 95% CI, 1.07–1.89 for RPR titer 1:16; OR, 5.16, 95% CI 2.99–8.89 for RPR titer > 1:32; Table 3). As shown in Figure 2, the odds ratios significantly increased with the increase of serum RPR titer (trend *p* < .001), which indicated artery stenosis was more closely correlated with increased serum RPR titer. Previous unknown or untreated syphilis was very common in our stroke center, which was independently associated with artery stenosis in seropositive stroke patients (OR, 3.63; 95% CI, 2.72–4.03; *p* < .001; Table 3). CRP levels were significantly higher for syphilis‐positive ischemic stroke patients compared with syphilis‐negative controls (1.95 ± 0.24 versus 1.68 ± 0.37, *p* < .001; Table 1), but not associated with stenosis in ischemic stroke patients with positive syphilis serology (OR, 1.24; 95% CI 0.62–2.50, *p* = .532; Table 3).

**Table 3:** Unadjusted and adjusted model for factors related to artery stenosis in the syphilis‐positive patients

Characteristics	No stenosis or artery stenosis < 50% (*n* = 426)	artery stenosis ≥ 50% (*n* = 242)	Univariate analyses			Multivariable analysis*		
			OR	95% CI	*p*‐value	OR	95% CI	*p*‐value
Untreated syphilis or syphilis of unknown (*n*, %)	53 (12.4)	64 (26.4)	2.50	1.64–3.79	<0.001	3.63	2.72–4.03	<0.001
hsCRP (mg/L)	1.95 ± 0.25	1.96 ± 0.2	1.21	0.60–2.44	0.578	1.24	0.62–2.50	0.532
Serum RPR titer								
0–1:2	215 (50.5)	97 (40.1)	0.65	0.47–0.92	0.014	0.66	0.45–0.98	0.053
1:4–1:8	76 (17.8)	31 (12.8)	0.68	0.43–1.09	0.114	0.91	0.53–1.53	0.724
1:16	60 (14.1)	38 (15.7)	1.14	1.02–1.80	<0.001	1.18	1.07–1.89	<0.001
≥1:32	75 (17.6)	76 (31.4)	2.12	1.49–3.10	<0.001	5.16	2.99–8.89	<0.001

Abbreviations: CI, confidence interval; hsCRP, hypersensitive C‐reactive protein; OR, odds ratio; RPR, rapid plasma regain.

* Multivariable analysis adjusted for age, sex, smoking, alcohol consumption, low physical activity, obesity, hypertension, diabetes mellitus, coronary heart disease, and history of previous stroke.

## 4 DISCUSSION

We found that ischemic stroke individuals with syphilis were distinct from the nonsyphilis population, with a younger age, fewer women, and a different risk factor profile. The relative younger patients in syphilis‐positive group, who had fewer traditional atherosclerosis risk factors, showed a tendency to be more prevalent in intracranial artery stenosis, MCA, and ACA in particular. Previous unknown or untreated syphilis and serum RPR titer ≥ 1:16 were independent risk factors for cerebral artery stenosis.

Today, syphilis is readily treated with penicillin, although is rapidly increasing in China (Chen et al., 2007; Yang et al., 2017). It is also a well‐recognized cause of stroke in young patients, particularly those originating from areas where syphilis is prevalent (Cordato et al., 2013; Liu et al., 2012). 1.88% of hospitalized ischemic stroke patients had positive syphilis serology in our stroke center, not associated with HIV co‐infection. The mechanisms by which syphilis infection alone confers increased risk of ischemic stroke are unclear and likely multifactorial. Our observation that most of the atherosclerosis risk factors for ischemic stroke were less prevalent among syphilis‐positive patients at baseline may indicate that there are additional syphilis‐specific mechanisms of increased stroke risk independent of traditional risk factors. Most studies assessed the extent of atherosclerosis by the presence of plaque and measuring IMT in carotid arteries. In our study, we did not find an association between carotid plaque, carotid IMT, and syphilis serology among ischemic stroke patients. However, estimating atherosclerosis in the extracranial portions of the carotid arteries is insufficient because atherosclerosis can develop anywhere throughout the cerebral arteries. Evaluating the degree of intracranial artery stenosis contributes to further explore the mechanism of syphilis vascular damage.

Based on clinical, radiologic analysis, we found that syphilis‐associated intracranial artery stenosis was a common etiology for ischemic stroke. The mechanism of syphilis‐induced stenosis is poorly understood but complex pathways of inflammation have been suggested (Czarnowska‐Cubala et al., 2013). The pathological characteristics of syphilitic vasculitis are meningeal and perivascular inflammatory cell infiltration, intimal chronic inflammatory fibrosis, ischemic necrosis, fibrosis of the tunica media, and intimal hyperplasia that can progress to stenosis or aneurysmal dilatation. However, obvious intracranial aneurysmal dilatation was rarely detected, and stenosis was common in the stroke patients living with syphilis based on our CTA or MRA findings. Our findings get support from some recent study that medium arteries respond with stenosis of the lumen and ischemic damage of dependent organs ensues when attacked by inflammation. The medium and small vessels are usually involved in neurosyphilis (Holland et al., 1986). The large arteries, such as aorta and its branches, are more likely to develop signs of wall destruction; manifesting as aneurysm formation, dissection or rupture (Frank et al., 1999; Weyand & Goronzy, 2013). A multitude of cytokines, all known as critical mediators in inflammation and immunity, has been identified in the vasculitic lesions (Weyand & Goronzy, 2013). High sensitivity c‐reactive protein (hsCRP), an important inflammatory marker, was not associated with stenosis in ischemic stroke patients with syphilis infection, although it was increased significantly compared with syphilis‐negative group in our study. Many cytokines appearing to serve different roles in the vasculitic process remain to be further identified.

On the other hand, it is speculated that syphilis‐related chronic inflammation lesions, within the vessel wall, not outside the vessel wall, may aggravate the process of atherosclerosis stenosis. It is intriguing that the pathogenic understanding of atherosclerosis has undergone a marked change recently. Extensive research evidences indicate that atherosclerosis, previously recognized as a lipid storage disease, is now emerging as an inflammatory process in which immune responses participate in every stage of the disease process (Back et al., 2019). Our results support and extend prior findings on syphilis‐induced stenosis, which is probably caused by aggravating the process of atherosclerotic stenosis in intracranial arteries.

Intracranial artery stenosis is more prevalent in Asians than in Westerners. Various explanations for the racial differences include genetic susceptibility, as well as differences in lifestyle and risk factor profiles. The ring finger protein 213 (RNF213) polymorphism was identified to be associated with intracranial artery stenosis in East Asian population, which was much rarer in non‐Asian populations (Kamimura et al., 2019; Okazaki et al., 2019). The incidence of syphilis has increased and remained at a fairly high level in China during the past 30 years. We found syphilis infection, especially when less well controlled (i.e., previous unknown, untreated syphilis, higher serum RPR titer), contributed to stroke risk independently of but in addition to traditional risk factors. We explored the correlation between the serum RPR titer and intracranial stenosis, and serum RPR titer ≥ 1:16 had been demonstrated as a risk factor for moderate‐to‐severe stenosis or occlusion of intracranial arteries. Recently, serum RPR titer was found to be an independent predictor for neurosyphilis. Our results get support from another more recent study that the patients with serum RPR titer ≥ 1:32 had more than 5‐fold increased risk of asymptomatic neurosyphilis (Cai et al., 2017). Many ischemic stroke patients with positive syphilis serology did not receive definitive syphilis treatment in the past, because most patients are asymptomatic or present with nonspecific symptoms before admission. It is presumed that the widespread use of antibiotics for infections unrelated to syphilis masks the symptoms of syphilis, but unfortunately chronic vascular damage persisted. Thus, the serologic tests for syphilis in ischemic stroke patients are essential and should be considered as part of the diagnostic work‐up of stroke in China. Recognizing and paying attention to syphilis‐associated intracranial stenosis contribute to the appropriate interventions of stroke according to etiology.

There were some limitations to the study. First, the inpatients recruitment may have been biased against milder cases in outpatient clinics. Second, the precise incidence of neurosyphilis remains unknown in this study, because most patients did not undergo lumbar puncture or had a nondiagnostic CSF finding. The absence of diagnosis of neurosyphilis limited our ability to further subdivide seropositive stroke patients into different etiologic subgroups. Last, our understanding of the pathologic mechanisms of syphilis‐related stenosis is incomplete. Further serologic and imaging tests should be used to check for the possible inflammatory cytokines and structure changes of artery wall.

## 5 CONCLUSIONS

With this retrospective cohort study, we found an association between positive syphilis serology and intracranial stenosis in ischemic stroke patients. Syphilis infection, especially when less well controlled, contributes to intracranial stenosis in ischemic stroke patients independently of traditional atherosclerotic risk factors.

## CONFLICT OF INTEREST

None declared.

## AUTHOR CONTRIBUTIONS

Wei Yue contributed to the concept initiation and study design. Lei Xiang and Tao Zhang contributed to data analysis and manuscript drafting. Biao Zhang, Chao Zhang, and Wanzhen Cui contributed to data acquisition.

### Peer Review

The peer review history for this article is available at https://publons.com/publon/10.1002/brb3.1906.

## Data Availability

The data that support the findings of this study are available on request from the corresponding author. The data are not publicly available due to privacy or ethical restrictions.
